# Discrepancies in Wechsler Adult Intelligent Scale III profile in adult with and without attention‐deficit hyperactivity disorder

**DOI:** 10.1002/npr2.12106

**Published:** 2020-04-25

**Authors:** Toshinobu Takeda, Youta Nakashima, Yui Tsuji

**Affiliations:** ^1^ Department of Clinical Psychology Ryukoku University Kyoto Japan; ^2^ Rakuwakai Otowa Hospital Department of Clinical Psychology Kyoto Japan; ^3^ Graduate School of Psychological Science Health Science University of Hokkaido Sapporo Japan; ^4^ Japan Society for the Promotion of Science Tokyo Japan

**Keywords:** adult attention‐deficit hyperactivity disorder, attention‐deficit hyperactivity disorder, comorbidity, discrepancy, WAIS

## Abstract

**Aim:**

The Wechsler Adult Intelligent Scale (WAIS) is the most frequently administered cognitive assessment for adult Attention‐deficit hyperactivity disorder (ADHD); therefore, identifying discrepancies in WAIS profile in patients and comparing with matched controls would be clinically and diagnostically beneficial.

**Methods:**

The WAIS‐III profiles of 50 adults with ADHD were compared to an age‐matched typical development (TD) group.

**Results:**

The adult ADHD group exhibited significantly lower WAIS‐III working memory (WM) and processing speed (PS) indices. However, these differences disappeared when intelligence quotient (IQ), Beck Depression Inventory (BDI) score, or Autism Quotient (AQ) score was included as a covariate. The adult ADHD group also demonstrated significantly lower scores in several WM‐ and PS‐domain subscales, while crystallized abilities were comparatively preserved. Additionally, only a small portion of participants in both groups lacked any significant gaps between WAIS‐III verbal and performance IQ scores (VIQ–PIQ) or associated indices.

**Discussion:**

This study confirms previous findings that adult ADHD patients have deficits in WM and PS. However, it is highly likely that comorbidities such as depression and autism spectrum disorder contribute to lower WM and PS scores in adult ADHD. Unexpectedly, a “flat profile” is uncommon even in TD adults. Therefore, clinician should assess how WAIS deficits affect daily life rather than merely considering an uneven WAIS profile when diagnosing and treating adult ADHD.

## Abstract 2

Although attention‐deficit hyperactivity disorder adults have lower scores of WM and PS in WAIS‐III, the comorbidities such as depression considerably contribute to these findings. Significant discrepancies in VIQ‐PIA and among index scores are common even in TD adults.

## Graphical abstract

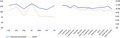

## 1 INTRODUCTION

Attention‐deficit hyperactivity disorder (ADHD) is characterized by inattention and hyperactivity‐impulsivity, which together have deleterious impacts on many facets of daily life. The estimated worldwide prevalence of adult ADHD is 2%‐5%.^1^, ^2^, ^3^

In childhood, main caregivers or teachers frequently first notice ADHD‐related behaviors and take children to see doctors, while adults suspecting ADHD seek treatment themselves or are advised by workplace supervisors, spouses, or parents. However, self‐referral may lead to under‐ or over‐diagnosis of adult ADHD.^4^ Barkley has criticized the reliance of childhood ADHD diagnosis on questionnaires from parents and/or teachers as circular reasoning.^5^ Diagnosing adult ADHD is also problematic as it must depend on the remote memory of self or by relatives. Ideally, the diagnostic process should comprise proxy ratings, interviews, clinical judgments, and objective assessment procedures as well as self‐reports,^6^ however, there are many cases in which clinicians cannot obtain such information. Although clinicians can obtain relevant behavioral information, there are often discrepancies between reports from different sources as is often the case in child ADHD.^7^ To avoid such difficulties in diagnosis and to reduce reliance on subjective reports, an external validator would be of great value.

The continuous performance test (CPT), eye tracking task, functional MRI, and event‐related potentials have demonstrated promise for detecting ADHD;^8^, ^9^, ^10^ (Walker, Shores, Trollor, Lee, & Sachdev, 2000)^11^ however, these tests require specialized equipment and are difficult to administer. Among clinical tools, the Wechsler Intelligent Scale is most frequently employed. For children with ADHD, several investigations of WISC‐III or ‐R profiles have constantly shown declines in Freedom from Distractibility and PS as index scores, and coding and digit span decline for subtests.^12^ Subsequently, a poor ACID (Arithmetic, Coding, Information, digit span) and SCAD (Symbol Search, Coding, Arithmetic, digit span) profiles were found in ADHD.^13^, ^14^, ^15^ However, these specific WAIS profiles for ADHD are based on findings in children and have not been confirmed in adult ADHD. One reason for this lack of formal investigation is that the WAIS profile of adult ADHD patients is more likely to be influenced by comorbidities than ADHD is in childhood.^16^ Another concern is uncertainty about the profile of adults with typical development (TD) as a comparator. Thus, diagnosis of developmental disorders based on variation in WAIS profile, especially among index scores, may be erroneous as it is unknown how frequent such variation is in TD.^17^, ^18^

The WAIS‐IV has been available in Japan since 2018. There are several changes from WAIS‐III to WAIS‐IV such as abolition of VIQ/PIQ, the introduction of General Ability Index (GAI), a minor change of digit span and the addition of visual puzzles to the perceptual reasoning index (PRI). Thus, it is a good opportunity to marshal data about ADHD profile of the former version of the WAIS (ie, WAIS‐III) so that these findings would be passed onto the next version of the WAIS (ie, WAIS‐IV).

In this study, the diagnostic utility of the WAIS‐III profile was investigated in adults with confirmed ADHD by comparing to WAIS‐III profiles to a large control cohort.

## 2 METHODS

### 2.1 Participants

This study involved 90 participants from two groups: (a) a group of 50 adults diagnosed with ADHD (27 males, age = 31.42 (9.78), age range = 19‐54) and (b) a control group of 40 adults (20 males, age = 31.48 (9.27), age range = 19‐53). The adult control group was recruited from Japanese companies and facilities or universities. The ADHD group was recruited from outpatients at the clinics where the first author was working.

Exclusion criteria for all participants were a current major depressive or manic‐depressive episode, history of psychosis, full‐scale IQ (IQ) < 80, sensory‐motor handicap, and neurological illness. Since many ADHD patients took WAIS‐III shortly after their first visit, only two of them had taken methylphenidate. They stopped taking ADHD medication on the testing day. No patient was prescribed atomoxetine.

The ADHD group comprised 32 individuals with predominantly inattentive presentation, 17 with combined, and one with predominantly hyperactive/impulsive presentation. Comorbidities in the present or the past in the ADHD group (some participants had more than one) were major depressive disorder (MDD, n = 13), dysthymic disorder (n = 13), autism spectrum disorder (ASD, n = 5), social anxiety disorder (n = 2), obsessive‐compulsive disorder (OCD, n = 2), generalized anxiety disorder (n = 1), tic disorder (n = 1), post‐traumatic stress disorder (PTSD, n = 1), substance use disorder (SUD, n = 1), and developmental coordination disorder (DCD, n = 1).

All the participants were administered the WAIS‐III, Assessment System for Individuals with ADHD (ASIA), and Mini International Neuropsychiatric Interview (MINI) and also answered the Japanese versions of the Beck Depression Inventory (BDI)^19^ and Autism Quotient‐short version (AQ).^20^ Participants also completed the Adult ADHD Self‐Report Scale (ASRS), an 18‐item questionnaire based on DSM‐IV diagnostic criteria.^21^

This study was approved by the Ryukoku university ethics committee. All participants gave written informed consent after a detailed description of study aims and protocols.

#### 2.1.1 ASIA

The ASIA is a Japanese semi‐structured diagnostic interview for adult ADHD. The ASIA ADHD criteria A, corresponding to DSM‐5 ADHD criteria A, is comprised of 144 questions that assess nine inattention symptoms and nine hyperactivity‐impulsivity symptoms in childhood and adulthood on a 3‐point scale (0 = *never*, 1 = *sometimes*, 2 = *often/always*). The ASIA ADHD criteria B to E, corresponding to DSM‐5 ADHD criteria B to E, are evaluated on a 2‐point scale (0 = *No*, 1 = *Yes*). The ASIA shows acceptable reliability and validity.^22^ Basically, diagnoses were made according to the ASIA. At the clinic, the first author administered the ASIS to make diagnosis of ADHD. The control subjects are convenient samples. We scored the results of their questionnaires and confirmed if they did not exceed the cutoff scores of the screener for ADHD, ASD, or depression.

#### 2.1.2 WAIS‐III

The WAIS‐III is an established intelligence test. It provides four index scores, verbal comprehension index (VC), perceptual reasoning index (PR), working memory index (WMI), and processing speed index (PSI) in addition to full intelligence quotient (FIQ), Verbal IQ (VIQ), and Performance IQ (PIQ). The VC includes vocabulary (V), similarities (S), and comprehension (C) subtests. The PR includes block design (BD), picture completion (PC), and matrix reasoning (MR) subtests. The WMI includes arithmetic (A), digit span (DS), and letter‐number sequencing (LN) subtests. Finally, the PSI includes coding (CD) and symbol search (SS) subtests.^23^

#### 2.1.3 MINI

The Mini‑International Neuropsychiatric Interview (MINI) is a structured interview used to diagnose 17 DSM disorders. It is composed of 130 questions with adequate psychometric properties for the English version. This measure was validated with a Japanese sample, with adequate levels of reliability and validity (Otsubo et al 2005).^24^

#### 2.1.4 BDI

The Beck Depression Inventory‐II (BDI‐II) is a self‐report inventory that evaluates depression severity. BDI‐II includes 21 items measuring depressive symptoms, such as sadness, pessimism, suicidal thoughts or wishes, tiredness or fatigue, loss of energy, and loss of pleasure. Each item is scored on a four‐point scale ranging from 0 to 3. Higher scores indicate higher depression severity. The Japanese version of the BDI‐II developed by Kojima et al^19^ has shown the same internal reliability and validity as the original version.

#### 2.1.5 AQ‐J

The AQ is a self‐reporting questionnaire for autism spectrum disorders, consisting of 50 items rated on 4‐point scale. It had satisfactory internal consistency reliability, test‐retest reliability and construct validity.

#### 2.1.6 ASRS‐J

The Adult ADHD Self‑Report Scale‐Japanese version (ASRS‑J) parallels the English version of the ASRS (Kessler et al 2007)^25^ and consists of 18 items assessing ADHD symptoms rated on a 5‐point scale (0 = never, 1 = rarely, 2 = sometimes, 3 = often, and 4 = very often). The ASRS‐J shows acceptable psychometric properties.^21^

### 2.2 Statistical analyses

IBM SPSS Statistic 25 was used for statistical analyses. Differences in frequencies were examined by chi‐square test unless the minimum case number (frequency) was 5 or below, in which case Fisher exact test was employed. Specific comparisons and tests are described below.

#### 2.2.1 Group differences in WAIS‐III scores and subscores

Full‐scale IQ, VIQ, PIQ, index scores (VC, PO, WM, PS), and index subscale scores were compared between adult ADHD and TD groups by independent samples *t* test with effect size (Cohen's *d*: |d| = 0.20, small; |d| = 0.50, medium, |d| = 0.80, large).^26^ If the *t* test indicated statistical significance (*P* < .05), ANCOVA was conducted with FIQ as a covariate, and then, ANCOVA with BDI or AQ as a covariate was conducted.

#### 2.2.2 Group differences in frequencies of significant discrepancy among WAIS‐III VIQ‐PIQ and index scores

Ratio gaps of significant discrepancy at the 15% or 5% levels in VIQ–PIQ or any 6 pairs of index scores between groups were evaluated by chi‐square test. Additionally, ratio gaps for index pairs in each discrepancy direction were compared between groups by chi‐square test (eg, for VC–WM, 5% positive discrepancy means VC > WM at the 5% level and negative 15% discrepancy means VC < WM at the 15% level). Furthermore, the ratios of indices with no discrepancy in VIQ‐PIQ or 6 pairs of index scores were tested by chi‐square test at 15% or 5% levels.

#### 2.2.3 Scatter

A range of 8 points or more among verbal or performance scales was considered abnormal scatter.^27^ The chi‐square test was administered to assess group differences in the frequencies of abnormal scatter.

#### 2.2.4 Absolute strengths and weaknesses compared to standardized data

To examine group differences in the frequencies of FIQ, VIQ, PIQ, and any 4 index scores over 115 or below 85 (ie, average ± 1 SD) or over 130 or below 70 (ie, average ± 2 SD), chi‐square test was performed. Similarly, chi‐square test was performed to examine group differences in the frequencies of subscale scores over 13 or below 7 (ie, average ± 1 SD).

#### 2.2.5 Relative strengths and weaknesses

For each subscale, significant deviation from each individual's (not population's) mean was assessed at the 15% and 5% level. If there was significant difference between VIQ and PIQ at ≥15% level, deviation of verbal (performance) scale scores from the mean verbal (performance) was checked. Then, the frequencies of relative strengths and weaknesses were compared between ADHD and TD groups.

#### 2.2.6 Predicting ADHD with WAIS variables

Logistic regression analysis with stepwise forward elimination and maximum likelihood estimation was performed to predict ADHD from WAIS variables. Only variables that reached significance in univariate comparison were entered in the model.

## 3 RESULTS

There was no significant group difference in mean age (*t* = 0.03, *P* = .98) or gender ratio (χ^2^ = 0.14, *P* = .83). The ADHD group demonstrated a significantly higher BDI‐II score than the TD group did [15.70 (10.76) vs 5.63 (6.02), *t* = 5.29, *P* = .000)], and a significantly greater proportion of the ADHD group exceeded the BDI‐II cutoff (14) for mild depression compared to the TD group (χ^2^ = 9.41, *P* = .002). Additionally, there was a moderate positive correlation between BDI‐II score and ASRS score (*r* = 0.56, *P* = .000).

Total ASRS score and both inattention and impulsivity‐hyperactivity subscores were significantly higher in the ADHD group than in the TD group (ASRS: *t* = 8.06, *P* = .00, inattention: *t* = 8.49, *P* = .00, impulsivity‐hyperactivity: *t* = 6.11, *P* = .00). The ADHD group also demonstrated a significantly higher AQ score than the TD group did (*t* = 6.10, *P* = .00), and there was a moderate correlation between AQ and ASRS score (*r* = 0.59, *P* = .00). Additionally, the proportion of subjects with AQ scores exceeding the cutoff (7) for ASD was significantly greater in the ADHD group than in the TD group (χ^2^ = 14.97, *P* = .000).

### 3.1 Group differences in WAIS‐III scores (FIQ, VIQ, PIQ, index scores, and subtest scores)

**Figure 1:**
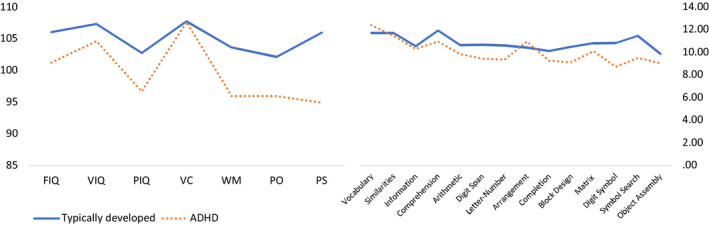
WAIS profile in ADHD and control. Arrangement, Picture Arrangement; Completion, picture completion; Digit Symbol, Digit Symbol Coding; FIQ, full intelligence quotient; Letter‐number, letter‐number sequencing; Matrix, matrix reasoning; PIQ, Performance intelligence quotient; PO, Perceptual organization; PS, Processing speed; VC, Verbal comprehension; VIQ, Verbal intelligence quotient; WM, Working memory

There were no group differences in FIQ, VIQ, PIQ, VC, and PO (FIQ: *t* = 1.67, *P* = .10; VIQ: *t* = 0.93, *P* = .36; PIQ: *t* = 1.95, *P* = .06; VC: *t* = 0.04, *P* = .97; PO: *t* = 1.91, *P* = .06) but the ADHD group exhibited significantly lower WM and PS index scores (WM: *t* = 2.34, *P* = .02; PS: *t* = 3.00, *P* = .000 by *t* test). However, the significance of the WM difference vanished when tested by ANCOVA with FIQ as a covariate (*F* = 2.78, *P* = .10). Furthermore, the significance of the PS difference was also reduced to marginal when compared by ANCOVA with BDI or AQ as a covariate (AQ: F = 2.17, *P* = .14, BDI: *F* = 3.72, *P* = .06). In subtests, the ADHD group demonstrated significantly higher coding and symbol search subscores (coding: *t* = 0.93, *P* = .04 symbol search: *t* = 2.75, *P* = .01) (see Tables 1 and 2 and Figure 1 for details).

**Table 1:** *t* Test of IQ, group index, and subscale

	ADHD (n = 50)	Control (n = 40)	*t*	Effect size (*d)*
FIQ	101.26 (13.90)	106.05 (13.20)	1.67	0.17
VIQ	104.60 (14.43)	107.30 (12.53)	0.93	0.09
PIQ	96.70 (14.81)	102.80 (14.70)	1.95	0.20
VC	107.64 (14.09)	107.75 (12.26)	0.04	0.00
PO	95.96 (14.56)	102.15 (16.08)	1.91	0.19
WM	95.96 (16.16)	103.63 (14.52)	2.34*	0.24
PS	94.92 (17.06)	106.00 (18.15)	2.98**	0.30
Vocabulary	12.88 (3.47)	11.70 (3.20)	‐0.96	0.34
Similarities	11.44 (2.96)	11.70 (2.96)	0.41	‐0.09
Information	10.26 (2.78)	10.53 (2.47)	0.47	‐0.10
Comprehension	10.94 (4.33)	11.90 (3.24)	1.20	‐0.25
Arithmetic	9.82 (3.41)	10.63 (2.70)	1.22	‐0.26
DS	9.42 (3.32)	10.65 (3.00)	1.82	‐0.39
LN	9.32 (3.25)	10.58 (3.04)	1.87	‐0.40
PA	10.94 (3.24)	10.40 (3.88)	‐0.72	0.15
PC	9.24 (2.84)	10.10 (3.40)	1.31	‐0.27
BD	9.10 (3.67)	10.45 (3.22)	1.83	‐0.39
MR	10.08 (3.16)	10.78 (2.64)	1.11	‐0.24
Coding	8.70 (3.66)	10.83 (3.34)	2.85**	‐0.61
SS	9.48 (3.10)	11.43 (3.63)	2.74**	‐0.58
OA	9.02 (3.24)	9.85 (3.68)	1.14	‐0.24

Note

Effect size (d): |*r*| ＝ 0.20 Small, |*r*| ＝ 0.50 Medium. |*r*| ＝ 0.80 Large.

Abbreviations: BD, Block Design; Coding, Digit Symbol Coding; FIQ, full intelligence quotient; LN, Letter‐Number Sequencing; MR, Matrix Reasoning; OA, Object Assembly; PA, Picture Arrangement; PC, picture completion; PIQ, Performance intelligence quotient; PO, Perceptual organization; PS, Processing speed; SS, Symbol Search; VC, Verbal comprehension; VIQ, Verbal intelligence quotient; WM, Working memory. *P<.05. **P < .01

**Table 2:** ANCOVA for WM and PS with FIQ, BDI, and AQ as covariates

	Covariate	ADHD (n = 50)	Control (n = 40)	*F*
WM	FIQ	95.96 (16.16)	103.63 (14.52)	2.27 n.s.
PS	FIQ	94.92 (17.06)	106.00 (18.15)	5.86*
	BDI	94.92 (17.06)	106.00 (18.15)	3.72 n.s.
	AQ	94.92 (17.06)	106.00 (18.15)	2.17 n.s.

Abbreviations: AQ, autism spectrum quotient; BDI, Beck Depression Inventory‐Second Edition; FIQ, full intelligence quotient; n.s., not significant, * PS, Processing speed; WM, Working memory.

*P*<.05.

### 3.2 Discrepancies between VIQ‐PIQ and associated indices

There was no significant difference in the rate of 5% or 15% VIP‐PIQ discrepancy between groups. However, the ADHD group showed significant VC‐WM discrepancy at 15% (χ^2^ = 9.02), VC‐WM discrepancy at 5% (χ^2^ = 9.51), VC > WM at 15% (χ^2^ = 6.77), VC < WM at 5% (χ^2^ = 9.50), VC > PS at 15% (χ^2^ = 4.94), and VC > PS at 5% (χ^2^ = 4.68) (all *P* < .05).

Not a single ADHD subject and only 3 TD subjects showed no discrepancy among indices at the 15% level (*P* = .08 by Fisher's exact test), while 4 TD and 2 ADHD subjects showed no discrepancy at the 5% level (*P* = .40 by Fisher's exact test) (see Table 3 for detail).

**Table 3:** Difference in significant discrepancy ration between ADHD and control (Fisher's exact test)

	ADHD (n = 50)	Control (n = 40)	χ^2^
VIQ‐PIQ			
15% level	31 (56.4%)	24 (43.6%)	0.04 n.s.
5% level	22 (52.4%)	20 (47.6%)	0.32n.s.
VC‐PO			
15% level	29 (58.0%)	21 (42.0%)	0.27 n.s.
5% level	23 (60.5%)	15 (39.5%)	0.65 n.s.
VC‐WM			
15% level	39 (67.2%)	19 (32.8%)	9.02**
5% level	35 (70.0%)	15 (30.0%)	9.51**
PO‐PS			
15% level	25 (54.3%)	21 (45.7%)	0.06 n.s.
5% level	22 (53.7%)	19 (46.3%)	0.11 n.s.
VC‐PS			
15% level	32 (58.2%)	23 (41.8%)	0.40 n.s.
5% level	29 (59.2%)	20 (40.8%)	0.57 n.s.
PO‐WM			
15% level	31 (54.4%)	26 (45.6%)	0.09 n.s.
5% level	23 (54.8%)	19 (45.2%)	0.02n.s.
WM‐PS			
15% level	28 (56.0%)	22 (44.0%)	0.01 n.s.
5% level	24 (54.5%)	20 (45.5%)	0.04 n.s.

Abbreviations: n.s., not significant; PIQ, Performance intelligence quotient; PO, Perceptual organization; PS, Processing speed; VC, Verbal comprehension; VIQ, Verbal intelligence quotient; WM, Working memory. **P < .01.

### 3.3 Group differences in score scatter

There were no significant group differences in abnormal V scatter (ADHD, 19; TD, 10: χ^2^ = 1.72, *P* = .19) or P scatter (ADHD, 16; TD, 7: χ^2^ = 2.46, *P* = .12).

### 3.4 Strengths and weaknesses in index and subscales scores according to standardized data

There were significant group differences in the frequencies of WM score under 85, PS score under 85, and PS score over 115 (WM under 85: χ^2^ = 6.11, *P* = .01; PS under 85: χ^2^ = 4.22, *P* = .04; PS over 115: χ^2^ = 5.61, *P* = .02). For subscales, there were significant differences in A under 7 (A_7_), D under 7 (D_7_), LN under 7 (LN_7_), PC over 13 (PC_13_), and CD under 7 (CD_7_) (A_7_: Fisher's exact test *P* = .04, D_7_: χ^2^ = 5.20, *P* = .02; LN_7_: Fisher's exact test *P* = .04; PC_13_: Fisher's exact test *P* = .02; CD_7_: χ^2^ = 8.01, *P* = .01).

### 3.5 Strengths and weaknesses in individual profiles

There were significant group differences in the ratios of C weakness (Cw) at 15%, D weakness (Dw) at 15%, PA strength (PAs) at 5% and 15%, PA weakness (PAw) at 5%, and MR strength (MRs) at 5% (Cw: χ^2^ = 6.11, *P* = .01; Dw: χ^2^ = 4.15, *P* = .04; PAs at 15% level χ^2^ = 6.45, *P* = .01; PAs at 5% level: χ^2^ = 5.56, *P* = .02; PAw: Fisher's exact test *P* = .04; MRs: χ^2^ = 4.36, *P* = .04).

### 3.6 Logistic regression analysis for prediction of adult ADHD

Finally, logistic regression analysis with stepwise forward elimination and maximum likelihood estimation was conducted, including variables that reached significance in previous analyses. If one variable was related to multiple significance discrepancies, the following rules were applied. 1. A continuous variable is preferred over a categorical variable. 2. Variables with larger effect sizes are preferred. Consequently, VC > PS (15%), A_7_, D_7_, LN_7_, PC_7_, and PAs (15%) were eliminated, while VC‐WM (5%), C_7_, MR_13_, and CD_7_ reached significance (VC‐WM: OR = 4.739, 95% CI = 1.713‐13.110, C_7_: OR = 6.955, 95% CI = 1.302‐37.147, MR_13_: OR = 5.854, 95% CI = 1.291‐26.536, CD_7_: OR = 0.867, 95% CI = 0.753‐0.998).

## 4 DISCUSSION

In our study, there was no significant difference in FIQ between ADHD and TD groups, but FIQ was numerically lower in the ADHD group. Theiling and Petermann speculated that this FIQ reduction is most likely due to decrements in WM and PS.^28^

Our study confirmed that these WM and PS deficits extend to adult ADHD. Alternatively, crystallized intelligence was preserved in adult ADHD, in accord with a previous study.^29^ Weakness in WM is consistent with Barkley's unified model of ADHD^5^ as an impairment in frontally mediated executive and attentional functions. On the other hand, many cognitive functions such as visual psychomotor speed and coordination, graphomotor abilities, cognitive flexibility, and attention are involved in PS.^30^ Notably, the significance of the WM deficit disappeared when controlling for FIQ, while the PS deficit remained when controlling for FIQ but vanished when controlling for BDI or AQ score. PS score would be influenced not only by ADHD‐specific deficits but by cognitive PS (which is impaired by depression), excessive concern for punctuality (a frequent feature of ASD and OCD), or clumsiness and poor coordination (a cardinal feature of ASD and DCD). Marchetta et al^31^ found that PS impairment in ADHD was not independent of comorbid disorders, suggesting that these deficits are not characteristics of ADHD per se. In the current study, 42% of the ADHD group exceeded the BDI cutoff score for mild depression and 48% exceeded the AQ cutoff for ASD, even though only five patients had a confirmed ASD diagnosis. Thus, it is highly likely that comorbid conditions contribute substantially to PS deficits in ADHD.

Compared to TD controls, the ADHD group also demonstrated significantly lower CD and SS subscores, both of which are included in the PS index. In adult ADHD, CD, DS, A, and BD are often impaired.^15^, ^32^ CD is included in all previously proposed ADHD profiles such as ACID, SCAD, and WDI. Thus, lower CD score is the most notable difference in the WAIS between ADHD patients and controls. However, clinicians should consider why PS score is low since CD is involved in many cognitive functions and is highly susceptible to comorbidities. In this study, the difference in SS (part of PS) also reached significance, however, SS is included only in SCAD. CD and SS both contribute to PS but are distinct in that CD depends on learning and SS on visual scan functions.^27^, ^33^

This study found no significant difference in VIQ‐PIQ discrepancy ratio between groups. While it is believed that differences between VIQ and PIQ are indicative of a cognitive disorder, this is not necessarily the case. The standardized sample for WAIS‐III showed that higher IQ individuals are more susceptible to large differences between VIQ and PIQ. This tendency is also found in discrepancies between index scores.

The ADHD group showed a significantly higher ratio of VC > WM and significantly lower ratio of VC < WM than the controls did. There are few studies reporting discrepancies in index scores among adults; however, children with ADHD do exhibit significant discrepancies between FD and other indices of the WISC‐III. Anastopoulous et al^34^ Since there was a significant difference in WM and PS, it is highly likely that there would also be significant discrepancies between certain WM‐ and PS‐related pairs (such as VC‐WM, VC‐PS, PO‐WM, PO‐PS).

The most striking finding in this study is the low “flat profile” rate in both TD and AHDH subjects. Only a small portion of both groups completely lacked discrepancies in index scores at the 15% or 5% level. The former was mathematically expected but the latter is beyond expectation. Significant discrepancies in index scores appear to be the rule rather than the exception and, thus, are of no value for ADHD diagnosis, although specific discrepancies may be clinically and diagnostically meaningful to ADHD deficits observed in daily life.

### 4.1 Strengths and weaknesses compared to standardized data (absolute strengths and weaknesses)

As expected, the frequencies of most individual scores exceeding the cutoff differed significantly between groups by *t* test. For instance, WM and PS index scores and CD subscales were absolute weaknesses in the ADHD group. Additionally, the ADHD group showed significantly higher rates of weakness in A, DS, and LN but a significantly higher rate of strength in PC compared to controls. However, these discrepancies could reflect deviation of control sample distributions from population distributions.

### 4.2 Strengths and weaknesses in individual profiles (relative strengths and weaknesses)

Strengths and weaknesses in individual profiles may be clinical useful for treatment decisions.^27^ In these analyses, the ADHD group demonstrated a significantly higher rate of weakness in C (15%) and DS (15%) and a higher rate of strength in PA (15% and 5%) and MR (15%). The strengths in PA and MR may be influenced by lower scores in PS‐related subtests (ie, CD and SS).

### 4.3 Scatter

In this study, 35% of ADHD and 20% of TD group participants showed abnormal scatter with no significant group difference in ratio of scatter. Ryan reported that intersubtest scatter among brain‐damaged patients as a whole was no greater than that for persons in the standardization sample.^35^ Since there was no significant difference between groups, the only conclusion possible based on these data is scatter among Verbal/Performance subtests indicates that the adult Verbal/Performance IQ represents a summary of diverse abilities rather than a unitary entity.^27^

### 4.4 Logistic regression

In this study, VC‐WM (5%), Cw (15%), MRs (15%), and CD_7_ reached significance in logistic regression analysis for discriminating adult ADHD from TD controls. The significance of Cw and MRs for distinguishing ADHD from TD controls is unexpected as neither has been identified previously as a diagnostic marker.^12^ Further study is needed to replicate the findings of the current study and to identify novel WAIS‐III score combinations that can reliably distinguish adult ADHD.

In accordance with the previous studies, scores of WM and PS are lower in adult ADHD than control; however, these deficits could be well explained by FSIQ, ASD trait, or depression. Unexpectedly, this study revealed that profile discrepancies in index scores (greater or smaller than expected) are the rule even in typically developed adult controls. Thus, it is risky for clinicians to diagnose ADHD or other developmental disorders, heavily relying on the up‐and‐down profile in index scores. This finding also raises the question of how most people adapt to the challenges posed by their environment despite an uneven WAIS profile.

Although there were some minor alterations in the WAIS‐IV from the WAIS‐III, findings in this study could be of clinical use because the WAISs are relatively robust among any versions. Theiling and Peterman^28^ reported that deficits identified with previous WAIS versions are robust in adults with ADHD. In combination with qualitative diagnostic information that measures real‐world problems, neuropsychological measures offer practical metrics of core deficits, strengths, and weaknesses are useful guides for psychoeducation and treatment.

Despite the minor alterations, the correlations between the IQ, index, and subtest scores in the former and the current Japanese version of WAIS are generally high (an adjusted correlation coefficient for FSIQ, index scores, and subtests are 0.86, 0.80‐0.87, 0.55‐0.87, respectively).^36^ Thus, it is legitimate to say that the WAIS is robust across versions. Moreover, General Ability Index which calculates the score based only on Verbal Comprehension Index and PRI is new to the WAIS and more suitable for ADHD patients than FSIQ because they have lower WS or PS score than control. Since the WAIS is the gold standard for comprehensive assessment of cognitive functions, clinicians and researchers should explore ways to optimize the utility of WAIS findings.

### 4.5 Limitations

This study has several limitations. First, the relatively small number of subjects precluded comparisons between genders and specific ADHD subtypes. Second, participants in both groups in this study are regarded as a convenient sample. Thus, this fact may limit generalizability of the results in this study. Finally, these findings do not necessarily reflect deficits in daily life. Test interpretation should consider ecological context,^33^ and many factors, including comorbidities (eg, learning disorders), educational, vocational, and sociocultural background, dexterity, eyesight, hearing ability, motivation, and familial values can affect WAIS results. Thus, clinicians should be aware that the testing situation is far different from ordinary daily life.

## CONFLICT OF INTEREST

The authors declare no conflicts of interest.

## AUTHOR CONTRIBUTIONS

TT and YT contributed to the study's design, acquisition of the data, analysis and interpretation of the data, and drafting of the manuscript. YN contributed to the acquisition of the data, the analysis, and interpretation of the data.

## INFORMED CONSENT

Written informed consents were obtained from all participants.

Registry and the Registration NO. of the study/trial: None.

Animal Studies: None.

## Data Availability

The data that support the findings of this study are not publicly available due to ethical restrictions. Specifically, informed consent for public data release was not obtained from the participants. The data are available on request from the corresponding author. Approval of the research protocol by an Institutional Reviewer Board: This study was approved by the ethical committee at the Ryukoku University.
